# Sociodemographic and clinical factors for non-hospital deaths among cancer patients: A nationwide population-based cohort study

**DOI:** 10.1371/journal.pone.0232219

**Published:** 2020-04-23

**Authors:** Qingyuan Zhuang, Zheng Yi Lau, Whee Sze Ong, Grace Meijuan Yang, Kelvin Bryan Tan, Marcus Eng Hock Ong, Ting Hway Wong

**Affiliations:** 1 Department of Supportive and Palliative Care, National Cancer Centre Singapore, Singapore, Singapore; 2 Policy Research and Evaluation Division, Ministry of Health, Singapore, Singapore; 3 National Cancer Centre Singapore, Singapore, Singapore; 4 Lien Centre for Palliative Care, Duke-National University of Singapore Medical School, Singapore, Singapore; 5 Saw Swee Hock School of Public Health, Singapore, Singapore; 6 Singapore General Hospital, Singapore, Singapore; 7 Duke-National University of Singapore Medical School, Singapore, Singapore; Osaka University Graduate School of Medicine, JAPAN

## Abstract

**Background:**

Factors associated with place of death inform policies with respect to allocating end-of-life care resources and tailoring supportive measures.

**Objective:**

To determine factors associated with non-hospital deaths among cancer patients.

**Design:**

Retrospective cohort study of cancer decedents, examining factors associated with non-hospital deaths using multinomial logistic regression with hospital deaths as the reference category.

**Setting/subjects:**

Cancer patients (n = 15254) in Singapore who died during the study period from January 1, 2012 till December 31, 2105 at home, acute hospital, long-term care (LTC) or hospice were included.

**Results:**

Increasing age (categories ≥65 years: RRR 1.25–2.61), female (RRR 1.40; 95% CI 1.28–1.52), Malays (RRR 1.67; 95% CI 1.47–1.89), Brain malignancy (RRR 1.92; 95% CI 1.15–3.23), metastatic disease (RRR 1.33–2.01) and home palliative care (RRR 2.11; 95% CI 1.95–2.29) were associated with higher risk of home deaths. Patients with low socioeconomic status were more likely to have hospice or LTC deaths: those living in smaller housing types had higher risk of dying in hospice (1–4 rooms apartment: RRR 1.13–3.17) or LTC (1–5 rooms apartment: RRR 1.36–4.11); and those with Medifund usage had higher risk of dying in LTC (RRR 1.74; 95% CI 1.36–2.21). Patients with haematological malignancies had increased risk of dying in hospital (categories of haematological subtypes: RRR 0.06–0.87).

**Conclusions:**

We found key sociodemographic and clinical factors associated with non-hospital deaths in cancer patients. More can be done to enable patients to die in the community and with dignity rather than in a hospital.

## Introduction

Cancer is a leading cause of death globally, accounting for an estimated 9.6 million deaths in 2018. [1] A large proportion of cancer deaths occur in hospitals for many developed countries. [2,3] Similar to global trends, cancer incidence is on the rise in Singapore; with cancer accounting for 30% of total population mortality. [4,5] Additionally, more than 50% of cancer decedents die in Singapore hospitals, [6,7] despite a majority patient preference for home deaths. [8–12]

Respecting preferences in terms of place of care and death is important. [13] Good cancer care includes a consideration of a patient’s needs, goals and preferences throughout their course of illness. [14] Respecting such preferences may provide better holistic well-being, increased peace and less intense grief for families. [15,16] Studies done in Singapore profiling end-of-life care preferences suggest most cancer patients prefer to die at home. [8–10,17] Such preferences remain relatively stable over trajectory of illness. [18]

Place of death is also a recognised quality indicator for end-of-life care. [19] Dying from cancer in hospitals is considered overly aggressive end-of-life care. [20–23] Costs for aggressive end-of-life care are substantially higher, driven by heavy dependence on hospitalizations. [24–28] Local data in Singapore suggests that hospitalizations are the largest driver of healthcare spending for oncology care. [29]

To develop services that effectively reduce hospital deaths, reduce costs and support dying in patients’ preferred place, understanding factors associated with non-hospital deaths in cancer patients is needed. These factors inform public health policies with respect to allocation of end-of-life care resources and tailoring supportive measures.

A systematic review of Western countries found 17 factors associated with place of death. Six factors were strongly associated with home deaths: low functional status, patient preferences for home death, use of home care, intensity of home care, living with relatives and extended family support. Conversely, non-solid tumours, ethnic minorities, previous admissions to hospitals and areas with greater hospital provision were associated with hospital deaths. [30] Literature specific to Asia suggest that marital status, poor functional status, having multidisciplinary home palliative care, lower caregiver burden and patient and family preferences increased the likelihood of dying at home. [31–35] Within Singapore, factors found to be associated with home death include age, female gender, Malay ethnicity, receipt of home palliative care, having a caregiver, non-cancer diagnosis, fewer prior hospitalizations and a preference for home death. [6,7,36]

While a recent systematic review concluded that low socioeconomic status increased the odds of hospital deaths, this conclusion was weaker for Asian countries due to a lack of published studies within this region. [37] To the best of our knowledge, local literature defining cancer specific risk factors for hospital deaths is also currently lacking; and remains critical in future identification of patients with unmet needs.

To meet the gap in literature, the primary objective of this study was to explore the influence of socioeconomic factors and clinical factors on places of death. We used the US National Cancer Institute’s Surveillance, Epidemiology, and End Results (SEER) categorisation of cancer types to increase granularity in examining cancer types. [38]

## Methods

### Setting, study design and participants

Cancer care in Singapore is predominantly provided within tertiary public institutions through a mixture of government subsidies, compulsory savings, compulsory national healthcare insurance and a state-provided “safety net”. [39] In recent years, to improve care across the cancer continuum, there have been increasing efforts to transition care to the community by empowering and increasing resources to community hospice providers. [40]

A retrospective national cohort study was conducted using state-wide administrative data of inpatient admissions, financial claims from the Ministry of Health (MOH) and death records from the Singapore Registry of Births and Deaths. [41]

Singapore residents who were discharged alive from hospital with a primary discharge diagnosis of cancer based on International Classification of Diseases codes (ICD-10-AM:C00 -C96) between January 1, 2012 and December 31, 2015, and a recorded death in the national death registry by December 31, 2015 were included in this study. Patients with unnatural deaths at sites such as roads (e.g. traffic accidents), ground floor of residential apartment blocks and reservoirs (possibly suicides) were excluded from the study (n = 52).

The STROBE guidelines were used for the reporting of this observational study. [42]

### Dependant variable

Place of death was as recorded in the patient’s death certificate and categorised as hospital, home, hospice and long-term care facilities (LTC)

### Independent variables

Socioeconomic variables were defined by three different aspects. First, we examined housing subsidy via mapping residential postal codes to housing type. Housing type (categorised by level of housing subsidy, ranging from private / non-subsidized housing, to intermediate subsidy with restrictions on resale and rental, to maximal subsidy, non-market housing) correlates with income status due to the public subsidized housing system in Singapore where income ceilings determine housing type eligibility. [43–45] Second, we calculated average monthly household income per capita percentiles based on the eligibility cut-off tiers for subsidized primary care under the Community Health Assist Scheme (CHAS). [46,47] Third, financial data on inpatient admissions paid from the government Medical Endowment Scheme (Medifund) was used. Medifund is a discretionary government-funded safety net to help the neediest Singaporeans with high post-subsidy inpatient bills, and takes into consideration the applicant’s financial, health and social circumstances. [48]

Clinical information was extracted from discharge diagnoses (age, gender, ethnicity, cancer sites, comorbidities) and admission records (length of stay, discharge disposition). We categorised ethnicity as Chinese, Malay, Indian and Others in accordance with the national approach towards racial categories. [49] Comorbid burden was computed using the Charlson Comorbidity Index (CCI). [50] Primary cancer sites were grouped by two-digit ICD-10-CM codes according to the SEER codes for cancers deemed to be single site primaries. [38] Metastases were grouped by three-digit SEER codes into brain, bone, lymph node, lung, liver, other gastrointestinal and other metastases. Status of home palliative care involvement was obtained from MOH Agency for Integrated Care records. [51]

The only variables with missing data were housing type (416, 2.72%) and ethnicity (144, 0.94%). Patients with missing variables were included in the analysis as “missing category”.

### Statistical analysis

Differences in mean of continuous variable by places of death were compared using Analysis of Variance. Corresponding differences in categorical variable were compared using chi-square test or Fisher’s exact test, as appropriate. Multivariable multinomial logistic regression analyses were used to estimate relative risk ratios (RRR) to examine the association between places of death and various covariates. Hospital death was used as the reference category and all independent variables listed above were included as covariates in the model. Sixty-six predictors were tested which was below the maximum number of predictors that could be fitted given total sample size and number of responses in each place of death category. [52] Model diagnostics were performed in which Wald tests were used to examine whether places of death categories could be combined, and spearman correlations were used to identify potential multicollinearity between independent variables. Sensitivity analyses were performed to examine the impact of outliers.

STATA version 13.0 (StataCorp) was used to perform statistical analysis. A two-sided *p*<0.05 was considered statistically significant.

### Research ethics and patient consent

This study was approved by SingHealth Central Institutional Review Board (CIRB Ref No: 2017/2908). Research and analysis were done on deidentified data. Waiver of requirement for informed consent was granted.

## Results

A total of 15254 decedents met the study eligibility criteria and were analysed. Within this cohort, 6.69% passing away in LTC, 14.38% in a hospice, 33.14% at home, and 45.79% in hospital. Mean (SD) age was 68 (13.6) years, majority were Chinese (81.05%) and 56.29% were male. Lung (21.40%), liver (9.39%), colon (9.37%), breast (7.28%) and stomach (6.14%) made up the top five solid organ malignancies, while 6.85% had haematological malignancies. Thirty-two percent received home palliative care before their deaths.

Table 1 and S1 Table summarises the distribution of each independent variable by places of death. Variables with small cell counts were reported as <5 to respect confidentiality of patients. There were distinct differences in sociodemographic characteristics of decedents by places of death. Decedents who passed away at hospital and hospice were younger than those who died at home and LTC. Malays and decedents who had home palliative care were more likely to pass away at home, while decedents with lower socioeconomic status (Medifund, <20^th^ income percentile, living in 1–2 room apartments) were less likely to do so.

**Table 1:** Sociodemographic and clinical characteristics of decedents by place of death.

Variable	No. (%)				P value
	Hospital	Home	Hospice	LTC	
Total	6985 (100.0)	5055 (100.0)	2194 (100.0)	1020 (100.0)	
Gender					
Male	4150 (59.41)	2543 (50.31)	1347 (61.39)	547 (53.63)	<0.001
Female	2835 (40.59)	2512 (49.69)	847 (38.61)	473 (46.37)	
Age, years					
<17	35 (0.50)	16 (0.32)	1 (0.05)	0 (0)	<0.001
17–34	118 (1.69)	44 (0.87)	18 (0.82)	2 (0.20)	
35–44	238 (3.41)	128 (2.53)	64 (2.92)	11 (1.08)	
45–54	848 (12.14)	430 (8.51)	242 (11.03)	86 (8.43)	
55–64	1756 (25.14)	1034 (20.45)	553 (25.21)	221 (21.67)	
65–74	1906 (27.29)	1286 (25.44)	614 (27.99)	274 (26.86)	
75–84	1567 (22.43)	1428 (28.25)	518 (23.61)	280 (27.45)	
≥85	517 (7.40)	689 (13.63)	184 (8.39)	146 (14.31)	
Mean (SD)	66.3 (13.7)	70.2 (13.6)	67.7 (12.7)	71.0 (12.2)	<0.001
Race					
Chinese	5565 (79.67)	3930 (77.74)	1973 (89.93)	896 (87.84)	<0.001
Indian	322 (4.61)	174 (3.44)	63 (2.87)	27 (2.65)	
Malay	678 (9.71)	647 (12.80)	88 (4.01)	54 (5.29)	
Others	345 (4.94)	260 (5.14)	55 (2.51)	34 (3.33)	
Missing	75 (1.07)	44 (0.87)	15 (0.68)	9 (0.88)	
CCI score					
0	3350 (47.96)	2318 (45.86)	1131 (51.55)	467 (45.78)	<0.001
1	1392 (19.93)	1129 (22.33)	436 (19.87)	196 (19.22)	
2	693 (9.92)	503 (9.95)	220 (10.03)	109 (10.69)	
3	489 (7.00)	373 (7.38)	155 (7.06)	77 (7.55)	
≥4	1061 (15.19)	732 (14.48)	252 (11.49)	171 (16.76)	
Medifund Use					
Yes	453 (6.49)	214 (4.23)	164 (7.47)	116 (11.37)	<0.001
No	6532 (93.51)	4841 (95.77)	2030 (92.53)	904 (88.63)	
CHAS Income Percentile					
<20th percentile	1983 (28.39)	1409 (27.87)	745 (33.96)	367 (35.98)	<0.001
20th-50th percentile	355 (5.08)	266 (5.26)	106 (4.83)	56 (5.49)	
>50th percentile	4647 (66.53)	3380 (66.86)	1343 (61.21)	597 (58.53)	
Housing Subsidy					
1–2 Room Apartment	331 (4.74)	187 (3.70)	233 (10.62)	130 (12.75)	<0.001
3 Room Apartment	1982 (28.38)	1351 (26.73)	743 (33.87)	287 (28.14)	
4 Room Apartment	2194 (31.41)	1655 (32.74)	608 (27.71)	280 (27.45)	
5 Room/Executive Apartment	1449 (20.74)	1171 (23.17)	351 (16.00)	169 (16.57)	
Private Housing^a^	836 (11.97)	612 (12.11)	193 (8.80)	78 (7.65)	
Missing	193 (2.76)	79 (1.56)	66 (3.01)	76 (7.45)	
Mean Length of Stay of Index Admission (SD), days	10.1 (13.6)	9.9 (12.1)	13.4 (16.2)	14.0 (21.5)	<0.001
Final Deposition of Index Admission					
Discharged	5989 (85.74)	4411 (87.26)	1541 (70.24)	606 (59.41)	<0.001
Transferred^b^	606 (8.68)	410 (8.11)	152 (6.93)	89 (8.73)	
Others^c^	390 (5.58)	234 (4.63)	501 (22.84)	325 (31.86)	
Home Palliative Care Involvement					
Yes	1872 (26.80)	2187 (43.26)	579 (26.39)	274 (26.86)	<0.001
No	5113 (73.20)	2868 (56.74)	1615 (73.61)	746 (73.14)	

Abbreviations: LTC, Long-Term Care Facilities; CCI, Charlson Comorbidity Index excluding cancer; Medifund, Medical Endowment Fund Scheme; CHAS, Community Health Assist Scheme.

a Included condominiums and landed properties

b To another tertiary healthcare institution

c Discharged against advice or abscondment

The results from our multinomial logistic regression model are presented in Table 2. Sensitivity analyses excluding outliers made no appreciable differences to the estimates of the model.

**Table 2:** Association of places of death with sociodemographic and clinical characteristics.

Variable	Relative Risk Ratio (95% CI)					
	Base category for places of death in multinomial logistic model = Hospital					
	Home	P value	Hospice	P value	LTC	P value
Gender (ref: Male)						
Female	**1.40 (1.28–1.52)**	**<0.001**	0.95 (0.84–1.07)	0.378	**1.27 (1.08–1.49)**	**0.005**
Age, years (ref: 55–64)						
< 17	0.65 (0.33–1.27)	0.207	**0.09 (0.01–0.70)**	**0.021**	*	-
17–34	**0.57 (0.39–0.84)**	**0.005**	0.61 (0.35–1.06)	0.078	**0.18 (0.04–0.74)**	**0.017**
35–44	0.82 (0.64–1.04)	0.102	1.02 (0.75–1.39)	0.911	**0.41 (0.22–0.77)**	**0.006**
45–54	**0.76 (0.66–0.88)**	**<0.001**	0.93 (0.78–1.12)	0.462	0.77 (0.59–1.01)	0.061
65–74	**1.25 (1.12–1.39)**	**<0.001**	1.01 (0.87–1.16)	0.943	1.15 (0.94–1.40)	0.174
75–84	**1.77 (1.58–1.98)**	**<0.001**	1.00 (0.89–1.17)	0.990	**1.39 (1.13–1.71)**	**0.002**
≥85	**2.61 (2.24–3.03)**	**<0.001**	1.06 (0.85–1.31)	0.604	**1.97 (1.51–2.56)**	**<0.001**
Race (ref: Chinese)						
Indian	0.88 (0.72–1.07)	0.199	**0.60 (0.45–0.80)**	**<0.001**	**0.49 (0.32–0.74)**	**0.001**
Malay	**1.67 (1.47–1.89)**	**<0.001**	**0.35 (0.28–0.45)**	**<0.001**	**0.49 (0.36–0.66)**	**<0.001**
Others	**1.24 (1.04–1.48)**	**0.016**	**0.50 (0.37–0.67)**	**<0.001**	**0.60 (0.41–0.88)**	**0.009**
CCI score (ref: 0)						
1	1.09 (0.99–1.21)	0.091	0.90 (0.78–1.03)	0.116	0.90 (0.74–1.08)	0.261
2	0.93 (0.81–1.06)	0.279	0.88 (0.73–1.05)	0.142	0.93 (0.73–1.18)	0.528
3	0.96 (0.82–1.12)	0.563	0.89 (0.72–1.09)	0.258	0.91 (0.69–1.20)	0.499
≥ 4	**0.86 (0.76–0.97)**	**0.012**	**0.67 (0.56–0.79)**	**<0.001**	0.89 (0.72–1.10)	0.275
Medifund Use (ref: No)						
Yes	**0.69 (0.58–0.83)**	**<0.001**	1.10 (0.90–1.35)	0.348	**1.74 (1.36–2.21)**	**<0.001**
CHAS Income Percentile (ref: > 50th percentile)						
20th - 50th percentile	0.95 (0.80–1.13)	0.559	0.99 (0.78–1.25)	0.924	1.22 (0.89–1.66)	0.209
< 20th percentile	0.92 (0.84–1.01)	0.065	1.06 (0.94–1.19)	0.332	1.05 (0.90–1.24)	0.512
Housing Type (ref: Private Housing^a^)						
1–2 Room Apartment	0.83 (0.66–1.03)	0.091	**3.17 (2.48–4.05)**	**<0.001**	**4.11 (2.96–5.72)**	**<0.001**
3 Room Apartment	0.95 (0.83–1.09)	0.488	**1.65 (1.37–2.00)**	**<0.001**	**1.52 (1.15–2.00)**	**0.003**
4 Room Apartment	1.07 (0.94–1.22)	0.301	**1.23 (1.02–1.49)**	**0.031**	**1.44 (1.09–1.89)**	**0.009**
5 Room/Executive Apartment	1.11 (0.97–1.27)	0.147	1.13 (0.92–1.39)	0.236	**1.36 (1.02–1.82)**	**0.037**
Length of Stay of Index Admission (per day increase)	1.00 (1.00–1.00)	0.939	**1.01 (1.00–1.01)**	**<0.001**	**1.00 (1.00–1.01)**	**0.033**
Final Disposition of Index Admission(ref: Discharged)						
Transferred^b^	1.00 (0.86–1.15)	0.951	0.93 (0.77–1.13)	0.484	**1.50 (1.17–1.92)**	**0.002**
Others^c^	**0.83 (0.70–0.99)**	**0.043**	**4.28 (3.66–5.01)**	**<0.001**	**6.57 (5.45–7.93)**	**<0.001**
Home Palliative Care (ref: No)						
Yes	**2.11 (1.95–2.29)**	**<0.001**	1.09 (0.97–1.22)	0.143	**1.23 (1.05–1.44)**	**0.011**
Primary Cancer Site^d^						
Other Head and Neck	0.49 (0.22–1.08)	0.076	0.49 (0.19–1.27)	0.142	1.68 (0.57–4.94)	0.348
Tongue	0.51 (0.26–1.00)	0.052	0.73 (0.34–1.56)	0.421	0.77 (0.25–2.34)	0.648
Oropharynx^&^	0.52 (0.21–1.32)	0.169	0.50 (0.17–1.47)	0.206	1.11 (0.30–4.16)	0.879
Nasopharynx	0.92 (0.57–1.48)	0.719	0.61 (0.32–1.16)	0.132	0.69 (0.28–1.69)	0.423
Hypopharynx^&^	0.97 (0.45–2.11)	0.945	0.65 (0.25–1.73)	0.392	0.19 (0.02–1.64)	0.131
Oesophagus	0.66 (0.41–1.08)	0.098	**0.40 (0.20–0.78)**	**0.007**	0.72 (0.30–1.71)	0.454
Stomach	0.93 (0.61–1.43)	0.755	0.57 (0.32–1.02)	0.060	0.79 (0.36–1.71)	0.545
Small Intestine	**0.44 (0.22–0.86)**	**0.016**	**0.28 (0.10–0.77)**	**0.014**	0.82 (0.25–2.67)	0.745
Colon	0.76 (0.50–1.15)	0.195	**0.55 (0.31–0.98)**	**0.043**	0.75 (0.35–1.59)	0.450
Rectosigmoid	0.81 (0.51–1.29)	0.378	0.60 (0.32–1.12)	0.110	0.77 (0.33–1.78)	0.541
Rectum	0.75 (0.48–1.17)	0.204	0.69 (0.38–1.26)	0.223	1.16 (0.53–2.57)	0.709
Anus^&^	1.60 (0.63–4.04)	0.324	2.26 (0.78–6.52)	0.132	2.83 (0.69–11.61)	0.148
Liver	0.84 (0.56–1.28)	0.426	**0.55 (0.31–0.98)**	**0.042**	0.79 (0.37–1.68)	0.534
Biliary	0.90 (0.56–1.45)	0.664	**0.51 (0.26–1.00)**	**0.050**	0.44 (0.17–1.15)	0.095
Pancreas	0.82 (0.53–1.26)	0.354	0.60 (0.33–1.08)	0.089	0.73 (0.33–1.61)	0.430
Other Facial	1.03 (0.48–2.18)	0.947	0.50 (0.15–1.39)	0.165	1.53 (0.46–5.12)	0.490
Larynx	0.60 (0.31–1.14)	0.118	0.53 (0.24–1.17)	0.117	0.76 (0.26–2.18)	0.611
Trachea and Lung	**0.61 (0.41–0.92)**	**0.018**	**0.54 (0.31–0.94)**	**0.030**	0.73 (0.35–1.53)	0.406
Thymus, Heart, Mediastinum^&^	**0.30 (0.12–0.75)**	**0.011**	**0.15 (0.03–0.69)**	**0.015**	1.21 (0.33–4.48)	0.771
Bone^&^	1.70 (0.73–3.98)	0.219	0.66 (0.18–2.44)	0.537	1.38 (0.24–7.92)	0.718
Skin	**0.55 (0.32–0.96)**	**0.037**	**0.38 (0.17–0.83)**	**0.015**	0.52 (0.18–1.47)	0.214
Mesothelioma, pleura	0.65 (0.34–1.26)	0.199	1.02 (0.46–2.28)	0.959	1.43 (0.49–4.18)	0.510
Other soft tissue, Sarcoma^&^	0.59 (0.30–1.16)	0.125	0.85 (0.38–1.91)	0.696	0.51 (0.13–2.08)	0.347
Retroperitoneum^&^	1.35 (0.67–2.76)	0.403	0.95 (0.35–2.58)	0.920	0.38 (0.04–3.27)	0.379
Breast	**0.58 (0.38–0.89)**	**0.013**	**0.43 (0.24–0.78)**	**0.006**	0.88 (0.41–1.90)	0.754
Female Genital^&^	0.67 (0.31–1.45)	0.307	0.63 (0.21–1.93)	0.416	0.90 (0.22–3.66)	0.878
Cervix	0.83 (0.50–1.36)	0.459	0.76 (0.38–1.49)	0.419	1.44 (0.60–3.43)	0.415
Uterus	0.61 (0.37–1.02)	0.058	0.51 (0.25–1.05)	0.066	1.41 (0.60–3.33)	0.435
Ovary	0.64 (0.40–1.01)	0.056	0.64 (0.34–1.22)	0.174	0.97 (0.42–2.25)	0.951
Male Genital^&^	0.66 (0.25–1.75)	0.402	0.98 (0.35–2.74)	0.973	1.08 (0.21–5.73)	0.925
Prostate	**0.57 (0.36–0.91)**	**0.017**	**0.49 (0.26–0.92)**	**0.027**	0.66 (0.29–1.49)	0.315
Kidney and Ureter	0.74 (0.47–1.16)	0.194	0.57 (0.31–1.06)	0.075	0.80 (0.35–1.80)	0.584
Bladder	0.71 (0.43–1.18)	0.187	**0.44 (0.22–0.90)**	**0.025**	0.91 (0.37–2.23)	0.834
Brain, Spine	**1.92 (1.15–3.23)**	**0.013**	1.71 (0.87–3.36)	0.118	**2.52 (1.02–6.18)**	**0.044**
Thyroid^&^	0.78 (0.41–1.50)	0.463	0.54 (0.21–1.37)	0.193	0.16 (0.02–1.37)	0.094
Adrenal and Endocrine^&^	1.43 (0.55–3.71)	0.463	1.22 (0.37–4.08)	0.744	*	-
Other misc. malignancies	1.42 (0.73–2.77)	0.300	1.81 (0.81–4.01)	0.147	1.38 (0.43–4.46)	0.586
Unspecified Site	0.85 (0.55–1.32)	0.462	0.60 (0.33–1.10)	0.102	0.61 (0.27–1.41)	0.248
Hodgkin's Disease^&^	0.23 (0.05–1.15)	0.074	*	-	*	-
Follicular NHL (nodular)^&^	0.87 (0.36–2.13)	0.760	0.16 (0.02–1.32)	0.088	0.44 (0.05–3.89)	0.462
Diffuse NHL	**0.57 (0.35–0.92)**	**0.022**	**0.41 (0.21–0.81)**	**0.010**	0.76 (0.32–1.81)	0.532
Peripheral and cutaneous TCL^&^	**0.27 (0.11–0.63)**	**0.003**	**0.18 (0.05–0.58)**	**0.004**	0.63 (0.17–2.31)	0.488
Other and unspecified NHL	**0.41 (0.22–0.76)**	**0.004**	**0.18 (0.07–0.49)**	**0.001**	0.42 (0.13–1.35)	0.145
Misc IPD^&^	0.74 (0.36–1.54)	0.422	0.45 (0.16–1.24)	0.122	0.14 (0.02–1.23)	0.076
Multiple myeloma and MPCN^&^	**0.28 (0.15–0.50)**	**<0.001**	**0.13 (0.05–0.36)**	**<0.001**	**0.06 (0.01–0.48)**	**0.008**
Lymphoid leukaemia^&^	**0.28 (0.13–0.61)**	**0.001**	*	**-**	**0.11 (0.01–0.95)**	**0.045**
Myeloid leukaemia	**0.35 (0.21–0.59)**	**<0.001**	**0.15 (0.07–0.33)**	**<0.001**	**0.22 (0.07–0.66)**	**0.007**
More than one primary site	**1.57 (1.09–2.25)**	**0.014**	1.47 (0.90–2.39)	0.125	1.29 (0.66–2.52)	0.456
Secondary Cancer Site^d^						
Lymph node metastases	0.99 (0.82–1.20)	0.897	**1.33 (1.02–1.72)**	**0.032**	1.13 (0.80–1.60)	0.486
Lung metastases	1.16 (0.96–1.40)	0.133	**1.49 (1.15–1.94)**	**0.002**	1.13 (0.80–1.60)	0.483
Gastrointestinal Metastases	**1.33 (1.08–1.64)**	**0.008**	1.30 (0.98–1.74)	0.073	1.07 (0.72–1.58)	0.748
Liver metastases	**1.37 (1.14–1.64)**	**0.001**	**1.78 (1.38–2.28)**	**<0.001**	1.14 (0.81–1.59)	0.455
Bone metastases	**1.25 (1.03–1.51)**	**0.021**	**1.46 (1.13–1.88)**	**0.004**	**1.54 (1.10–2.16)**	**0.011**
Brain metastases	**2.01 (1.62–2.49)**	**<0.001**	**2.53 (1.91–3.34)**	**<0.001**	**1.86 (1.28–2.70)**	**0.001**
Other metastases	1.00 (0.87–1.16)	0.986	1.07 (0.89–1.28)	0.487	1.03 (0.80–1.33)	0.455
More than one secondary site	0.94 (0.81–1.09)	0.408	**0.77 (0.62–0.95)**	**0.013**	0.89 (0.68–1.17)	0.408

Abbreviations: LTC, Long-Term Care Facilities; ref, reference category; CCI, Charlson Comorbidity Index excluding cancer; Medifund, Medical Endowment Fund Scheme; CHAS, Community Health Assist Scheme; NHL, non-Hodgkin's lymphoma; TCL: T-cell lymphomas; misc, miscellaneous; IPD, immunoproliferative diseases; MPCN, malignant plasma cell neoplasms.

a Included condominiums and landed properties

b To another tertiary healthcare institution

c Discharged against advice or abscondment

d Refer to Table 1 for the ICD-10-CM topography codes for each category of cancer site

*Not estimable as there were no decedent in the category

Note: RRR estimates in bold are significantly different from value 1 (p<0.05).

^&^ Caution with interpretation due to low cell counts

### Home death vs hospital death

Independent factors associated with higher risks of dying at home than in hospitals were increasing age, females, Malay and Other ethnicities, receipt of home palliative care, being non-Medifund aided and having less comorbidities. (Table 2)

### Hospice death vs hospital death

Independent factors associated with higher risks of dying in hospice than hospitals were Chinese, having fewer comorbidities, living in smaller subsidized housing types and being discharged against advice or abscondment during index hospitalisation. (Table 2)

### Long-Term Care (LTC) death vs hospital death

Independent factors associated with higher risks of dying in LTC than hospitals were older age, females, Chinese, Medifund recipients, living in smaller subsidized housing types and being transferred to another tertiary institute, discharged against advice or absconded during index hospitalisation. (Table 2)

### Cancer-specific risk factors for hospital deaths and out-of-hospital deaths

Patients with solid organ tumours such as Breast, Prostate, Lung and other rarer sites had increased risk of dying in hospitals rather than home or hospice. Most haematological malignancies had higher risk for dying in hospital.

Primary brain malignancy and having metastatic cancer (e.g. brain metastases, bone metastases) were associated with increased risk for out-of-hospital deaths. (Table 2)

## Discussion

In this study, a large proportion of cancer patients (45.64%) died in hospitals while only 33.03% died at home and 21.07% in LTC/hospice. Like previous local studies, we confirm older age, female, Malay ethnicity and home palliative care involvement to be associated with home deaths. [6,7] Additionally, we found that primary brain cancer, metastatic disease and non-Medifund patients were more likely to die at home.

From our analysis, low SES patients (smaller housing types, Medifund recipients) were more likely to pass away in LTC or hospices rather than hospitals. Being discharged against advice or abscondment from hospital, which we classify as high-risk behaviours with possible underlying social and financial needs, was also strongly associated with LTC and hospice deaths. This contrasts with a recent systematic review that included studies mostly from the US, Canada and Europe suggesting that low socioeconomic position is a risk factor for hospital deaths. [37] We hypothesize that within Singapore’s healthcare system, early transfers to LTC or hospice were taking place for low SES patients when they lose the ability to self-care, resulting in higher proportions of death within these institutions. [53] While palliative care services are routinely provided within hospices, many LTC facilities are still unable to provide good quality palliative care services due to manpower and resource constraints and lack of training [40] Palliative care provision has to be strengthened within LTC to meet the needs of the socially disadvantaged who are more likely to die in such facilities. [54–56]

We found home palliative care involvement to be associated with increased likelihood of home or LTC deaths. This concurs with meta-analysis evidence that home palliative care increases the likelihood of dying at home. [57] Our findings reaffirm ongoing national efforts in improving capacity of community palliative care to meet the needs of patients and facilitating out-of-hospital-deaths. [40]

We found positive association between haematological malignancies and dying in hospital, echoing findings from studies done by western counterparts. [30,58–60] Additionally, this association remained strongly significant for almost all types of haematological malignancies. We postulate this is due to characteristics of underlying disease and treatment, including uncertain trajectories, indistinct transitions, prognostic difficulties and difficult symptoms (e.g. overwhelming sepsis, symptomatic anaemia, etc). [61] Referrals to palliative care occur less frequently for patients with haematological malignancies and often late in the disease trajectory, with many still undergoing aggressive treatment. [62] More research is needed to improve end-of-life outcomes for this group of patients.

Lastly, ethnicity was associated with place of death, suggesting that unmeasured sociocultural differences in perspectives influence utilization of hospice/LTC facilities. Malays were more likely to die at home, congruent with previous studies, possibly due to strong family and intergenerational support as well as religious beliefs. [63,64] In contrast, Indian and “other” (non-Chinese, non-Malay, non-Indian) minority ethnicities, compared to the Chinese, were more likely to die in hospital than in hospice or LTC. Additional studies with qualitative methodology may shed further light on this finding.

### Strengths and limitations

A key strength of our study is the linkage of nation-wide clinical data with socioeconomic profiles and healthcare utilization data. Analysing place of death outcomes as a multiclass problem prevents oversimplification to a binary outcome of “hospital” vs “others” as individuals may prefer to die in other settings and understanding the factors influencing each is important. Additionally, only a small percentage of our cohort had missing data.

One key limitation was the inability to capture important variables such as patient preferences for place of death, acute hospital utilization at end-of-life, cancer stage at death, health and function trajectories and additional socioeconomic variables such as employment status, education level and caregiver burden. As this study was limited to public hospitals, we could not capture those who received treatment solely in private centres. However, majority of healthcare in Singapore is provided by public hospitals so the effect may be minimal. [65] While some of the patients in our study may have died of other unrelated causes, this was mitigated by adjustment for comorbidity index at the time of index admission. Moreover, 91.26% of our cohort passed away from cancer as primary cause of death.

Finally, due to cancer epidemiology and the relatively small population in Singapore, the rarer cancers (e.g. bone, anus, female and male genital, Hodgkin’s disease) had low counts and hence the related statistics must be interpreted with caution.

### Implications and generalisability

Results from our study suggest directions for future studies and healthcare policies. Firstly, low SES patients are more likely to die in LTC or hospice than hospitals. Provision of good quality palliative care should expand towards LTC to meet the needs of socially disadvantaged patients who are more likely to die in such facilities. Secondly, patients on home palliative care were more likely to pass away at home or in LTC, reaffirming efforts on improving capacity of community palliative care to meet the needs of patients and facilitating out-of-hospital-deaths. Thirdly, if patients with haematological cancers are more likely to pass away in hospitals, then it is essential that adequate care is available within the hospital setting. Additionally, research is needed on their care preferences, reasons for hospital deaths and mitigation strategies if home death is preferred by these patients.

Considering the similarity of some of our study findings to international studies, the findings may be generalizable to other urban settings. However, culture and system-specific factors found in our study highlight the complexities of place of death.

## Conclusion

We found in this study key sociodemographic and clinical factors associated with non-hospital deaths among cancer patients. We believe our findings have implications for future policy making. High-risk groups for dying in hospitals may benefit from targeted models of care while better support can be tailored for those who pass away out of hospitals.

## Supporting information

S1 Table Cancer sites of decedents by place of death. (DOCX)
