# Supplementary material for: Sociodemographic and clinical factors for non-hospital deaths among cancer patients: A nationwide population-based cohort study
Source: PLoS One. 2020 Apr 23;15(4):e0232219. doi: 10.1371/journal.pone.0232219 (PMC7179880; doi:10.1371/journal.pone.0232219)
Supplement: S1 Table — (DOCX) [file pone.0232219.s001.docx]

Supplementary Table 1: Cancer sites of decedents by place of death

| Variable | ICD-10-CM  topography code | No. (%) | | | | P  value |
| --- | --- | --- | --- | --- | --- | --- |
|  |  | Hospital | Home | Hospice | LTC |  |
| Total |  | 6985 (100.0) | 5055 (100.0) | 2194 (100.0) | 1020 (100.0) |  |
| Primary Site: |  |  |  |  |  |  |
| Other Head and Neck | 00,03-06,14 | 31 (0.44) | 12 (0.24) | 8 (0.36) | 8 (0.78) | 0.052 |
| Tongue | 01-02 | 51 (0.73) | 18 (0.36) | 19 (0.87) | 6 (0.59) | 0.025 |
| Oropharynx | 09-10 | 22 (0.31) | 8 (0.16) | 6 (0.27) | <5 | 0.321 |
| Nasopharynx | 11 | 135 (1.93) | 88 (1.74) | 50 (2.28) | 16 (1.57) | 0.388 |
| Hypopharynx | 12,13 | 22 (0.31) | 16 (0.32) | 9 (0.41) | <5 | 0.539 |
| Oesophagus | 15 | 148 (2.12) | 83 (1.64) | 35 (1.60) | 20 (1.96) | 0.193 |
| Stomach | 16 | 382 (5.47) | 380 (7.52) | 120 (5.47) | 54 (5.29) | <0.001 |
| Small Intestine | 17 | 41 (0.59) | 19 (0.38) | 6 (0.27) | 5 (0.49) | 0.182 |
| Colon | 18 | 598 (8.56) | 528 (10.45) | 212 (9.66) | 92 (9.02) | 0.006 |
| Rectosigmoid | 19 | 149 (2.13) | 131 (2.59) | 61 (2.78) | 25 (2.45) | 0.233 |
| Rectum | 20 | 199 (2.85) | 153 (3.03) | 82 (3.74) | 42 (4.12) | 0.047 |
| Anus | 21 | 9 (0.13) | 16 (0.32) | 10 (0.46) | <5 | 0.025 |
| Liver | 22 | 667 (9.55) | 496 (9.81) | 184 (8.39) | 86 (8.43) | 0.175 |
| Biliary | 23-24 | 115 (1.65) | 107 (2.12) | 36 (1.64) | 11 (1.08) | 0.067 |
| Pancreas | 25 | 355 (5.08) | 307 (6.07) | 126 (5.74) | 45 (4.41) | 0.044 |
| Other Facial | 30-31,69 | 23 (0.33) | 18 (0.36) | 5 (0.23) | 6 (0.59) | 0.436 |
| Larynx | 32 | 48 (0.69) | 23 (0.45) | 17 (0.77) | 8 (0.78) | 0.265 |
| Trachea and Lung | 33-34 | 1465 (20.97) | 1011 (20.00) | 548 (24.98) | 240 (23.53) | <0.001 |
| Thymus, Heart, Mediastinum | 37-38 | 28 (0.40) | <5 | <5 | <5 | 0.012 |
| Bone | 40-41 | 16 (0.23) | 15 (0.30) | <5 | <5 | 0.780 |
| Skin | 43-44,46 | 73 (1.05) | 41 (0.81) | 16 (0.73) | 8 (0.78) | 0.400 |
| Mesothelioma, pleura | 45,384 | 37 (0.53) | 24 (0.47) | 17 (0.77) | 8 (0.78) | 0.322 |
| Other soft tissue, Sarcoma | 47,49 | 41 (0.59) | 22 (0.44) | 17 (0.77) | <5 | 0.197 |
| Retroperitoneum | 48 | 21 (0.30) | 26 (0.51) | 8 (0.36) | <5 | 0.119 |
| Breast | 50 | 521 (7.46) | 363 (7.18) | 132 (6.02) | 94 (9.22) | 0.010 |
| Female Genital | 51-52,57.7-57.9 | 21 (0.30) | 16 (0.32) | 5 (0.23) | <5 | 0.935 |
| Cervix | 53 | 101 (1.45) | 84 (1.66) | 34 (1.55) | 21 (2.06) | 0.467 |
| Uterus | 54-55 | 106 (1.52) | 70 (1.38) | 23 (1.05) | 22 (2.16) | 0.093 |
| Ovary | 56,57.0-57.4 | 172 (2.46) | 132 (2.61) | 52 (2.37) | 28 (2.75) | 0.879 |
| Male Genital | 60,62-63 | 17 (0.24) | 7 (0.14) | 8 (0.36) | <5 | <0.001 |
| Prostate | 61 | 209 (2.99) | 135 (2.67) | 58 (2.64) | 31 (3.04) | 0.667 |
| Kidney and Ureter | 64-66,68 | 194 (2.78) | 148 (2.93) | 69 (3.14) | 31 (3.04) | 0.819 |
| Bladder | 67 | 138 (1.98) | 110 (2.18) | 31 (1.41) | 24 (2.35) | 0.148 |
| Brain, Spine | 70-72 | 74 (1.06) | 98 (1.94) | 53 (2.42) | 22 (2.16) | <0.001 |
| Thyroid | 73 | 32 (0.46) | 28 (0.55) | 9 (0.41) | <5 | 0.259 |
| Adrenal and Endocrine | 74-75 | 11 (0.16) | 11 (0.22) | 6 (0.27) | 0 (0) | 0.453 |
| Other misc malignancies | 07-08,26,58,76 | 25 (0.36) | 35 (0.69) | 24 (1.09) | 6 (0.59) | 0.001 |
| Unspecified Site | 80 | 184 (2.63) | 190 (3.76) | 73 (3.33) | 26 (2.55) | 0.003 |
| Hodgkin's Disease | 81 | 13 (0.19) | <5 | 0 (0) | 0 (0) | <0.001 |
| Follicular NHL (nodular) | 82 | 15 (0.21) | 11 (0.22) | <5 | <5 | <0.001 |
| Diffuse NHL | 83 | 167 (2.39) | 79 (1.56) | 31 (1.41) | 19 (1.86) | 0.002 |
| Peripheral and cutaneous TCL | 84 | 47 (0.67) | 8 (0.16) | <5 | <5 | <0.001 |
| Other and unspecified NHL | 85 | 69 (0.99) | 24 (0.47) | 6 (0.27) | 5 (0.49) | <0.001 |
| Misc IPD | 88,93-96 | 30 (0.43) | 17 (0.34) | 7 (0.32) | <5 | 0.381 |
| Multiple myeloma and MPCN | 90 | 105 (1.50) | 28 (0.55) | 6 (0.27) | <5 | <0.001 |
| Lymphoid leukaemia | 91 | 72 (1.03) | 11 (0.22) | 0 (0) | <5 | <0.001 |
| Myeloid leukaemia | 92 | 189 (2.71) | 52 (1.03) | 13 (0.59) | 6 (0.59) | <0.001 |
| More than one primary site |  | 257 (3.68) | 218 (4.31) | 74 (3.37) | 38 (3.73) | 0.182 |
| Secondary Site: |  |  |  |  |  |  |
| Lymph node metastases | 77 | 1586 (22.71) | 1115 (22.06) | 560 (25.52) | 248 (24.31) | 0.008 |
| Lung metastases | 78.0-78.3 | 1406 (20.13) | 1148 (22.71) | 565 (25.75) | 240 (23.53) | <0.001 |
| Gastrointestinal metastases | 78.4-78.6,78.8 | 651 (9.32) | 606 (11.99) | 223 (10.16) | 90 (8.82) | <0.001 |
| Liver metastases | 78.7 | 1092 (15.63) | 1015 (20.08) | 501 (22.84) | 179 (17.55) | <0.001 |
| Bone metastases | 79.5 | 1063 (15.22) | 845 (16.72) | 437 (19.92) | 220 (21.57) | <0.001 |
| Brain metastases | 79.3 | 393 (5.63) | 438 (8.66) | 261 (11.90) | 104 (10.20) | <0.001 |
| Other metastases | 79.0-79.2,79.4, 79.6-79.8 | 535 (7.66) | 421 (8.33) | 221 (10.07) | 94 (9.22) | 0.003 |
| More than one secondary site | - | 1719 (24.61) | 1450 (28.68) | 750 (34.18) | 300 (29.41) | <0.001 |

Abbreviations: LTC, Long-Term Care Facilities;NHL, non-Hodgkin's lymphoma; TCL: T-cell lymphomas; misc, miscellaneous; IPD, immunoproliferative diseases; MPCN, malignant plasma cell neoplasms.

*Small cell counts are reported as <5 to respect confidentiality of patients. However we used the actual counts in our multinomial logistic regression model.
